# Automation in multi-dimensional gas chromatography

**DOI:** 10.1155/S146392468500027X

**Published:** 1985

**Authors:** G. M. Ogle, S. W. S. McCreadie, D. F. K. Swan

**Affiliations:** Pye Unicam Ltd York Street Cambridge CB1 2PX UK


Journal of Automatic Chemistry/*Journal of Clinical Laboratory Automation*, Vol. 7, No. 3 (Jul–Sep 1985), pp. 130–135

### Automation in multi-dimensional gas chromatography

**G. M. Ogle, S. W. S. McCreadie and D. F. K. Swan**
*Pye Unicam Ltd, York Street, Cambridge CB1 2PX, UK*

### Introduction

Few inventions have revolutionized the laboratory analyst's job as much as the microprocessor. It has been incorporated into nearly all laboratory equipment and instrumentation and the benefits have been widely felt; particularly so in the field of automatic chemistry. The requirements of automatic chemistry are that the instrument must be left to run automatically without operator attention: a task to which microprocessors are ideally suited. Not only can they control the entire analytical system, but, because they can act 'intelligently' with it, they can correct any deviation or errors detected.

Gas chromatography has experienced a leap in performance due to the microprocessor with oven control performance; has reached previously undreamt of levels. Simple electronic integrators were replaced by powerful microprocessor-based data-handling systems, which could simultaneously receive data from more than one source and could control the chromatographs linked into the analytical system. Automatic sampling and injection devices changed from being 'dumb' mechanical machines, which received instructions from punched tape or timers, to sophisticated, dedicated robots capable of flexibility, intelligence and a high degree of accuracy and repeatability.

With all of the instruments and many accessories being microprocessor controlled, it became possible to link them all together, and, by computer data links, to create a very powerful analytical system. Unfortunately, however, these complete analytical systems were very complex and definitely 'user unfriendly'. A system may consist of a main oven unit, a satellite chromatograph, automatic injection system, data handling, disk and graphics capability and be required to output data to an external computer for archiving. Such systems would often have a keyboard on each part of the set-up and could require many hours of work to start up, only to find that the system 'crashed' because one of the units was incorrectly programmed.

Perhaps a more serious problem was that with the emphasis being placed on the electronics side of the package, the chromatography, which is really the heart of the system, was ignored or given second place in the development budget.

The need for large, integrated GCs has always been very important, particularly for automated systems which must be left to run, often for days at a time. It was with this need in mind that the new PU4900 GC was developed and launched in late 1984.

The PU4900 is a new style of total analytical chromatograph that contains chromatography, data handling, disks and graphics and control functions of a satellite chromatograph and up to two autojectors within the basic package. All control functions and softwate manipulations are performed on a single QWERTY keyboard.

The systems design is such that the flexibility of manual operation has not been lost in the automatic mode and this can be illustrated by looking at a technique which is rapidly growing in popularity: capillary multidimensional chromatography, specifically the automatic aspects.

### Multi-dimensional chromatography

Multi-dimensional chromatography (MDC) is a technique in which it is possible to have two or more columns, normally of different stationary phases, within the same analytical system, and, by controlling gas pressures and flows, it is possible to decide the path of a sample through the system.

There are three main techniques used – heart cutting, back flushing and column switching. These may be used singly or in combination. Later in this article an application of heart cutting will be examined; it is convenient at this point to illustrate a heart cutting system.

A heart cutting arrangement allows an initial separation to be achieved on a first column, but, as the components of interest are eluting, rather than going into a detector, they are passed onto a second column where further separation is possible. Heart cutting therefore allows the analyst to 'cut' a part of a chromatogram, the 'heart', onto a second column. The unwanted components are vented to waste.

A simple block diagram of a heart cut system is illustrated in figure 1. A sample is injected with the system in position 1, but after time $t_1$, the system is switched to position 2. This allows material eluting from column 1, after time $t_1$, to be passed onto the second column, column 2. When the final piece of the 'heart' has eluted at time $t_2$, the system is switched back to position 1 and all material now eluting from column 1 is vented. While the unwanted material is being vented, the 'heart' is continuing chromatography on column 2. Column 2 is normally of a different stationary phase and consequently its

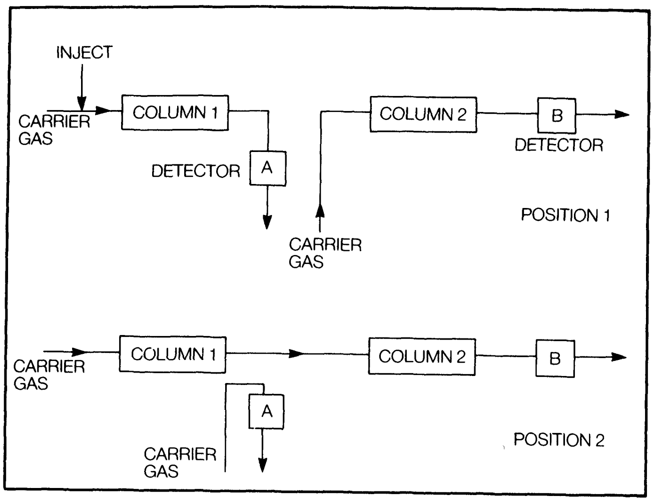

*Figure 1. Block diagram of a heart cut system.*

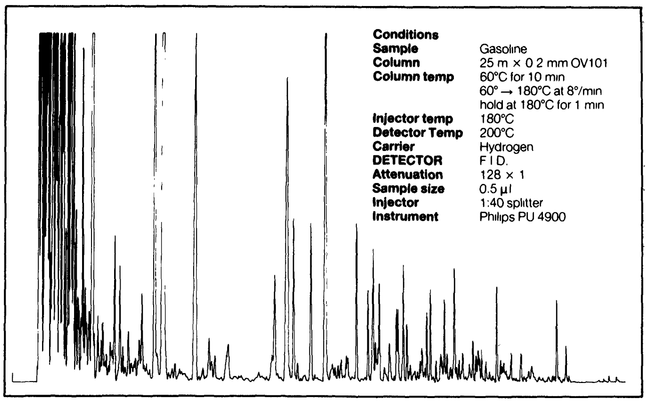

*Figure 2. Capillary chromatogram of petrol.*

separation properties are different from column 1. The reason that a two column system is used, rather than just column 2, is to achieve an initial separation of the complex mixture. Analysis of the mixture on column A or B results in a complex chromatogram in which the compounds of interest are obscured by co-eluting peaks; but it is unlikely that these peaks will be the same on both columns. In other words, the peak of interest, A, co-elutes from the first column with peak X, but by passing this mixture onto a different stationary phase, it is possible to achieve full resolution of A and X. If the second phase column had been used first, then A now co-elutes with Y and passage onto the other phase also achieves resolution.

That is the basic theory of a heart cutting MDC but how does it fit into the general analytical technique of GC? In the early 1960s, packed column chromatography was rapidly advancing but it reached a stage where the separating power of the column was the limiting factor. It was found that by coupling two columns together it was possible to achieve greatly increased resolution: the number of effective theoretical plates of the total system could be measured in tens of thousands.

When capillary columns became widely available in the early1970s, a single column could now have an effective plate number of $10^5$ and for some time MDC was largely ignored. Capillary columns have very good resolving power and can nearly always produce a separation of a mixture, but for very complex natural samples, such as hydrocarbon mixtures and essential oils, it is impossible to get base-line resolution of all components.

A typical capillary chromatogram of a light hydrocarbon fraction, petrol, is shown in figure 2. The profusion of peaks is particularly dense in the early part of the chromatogram where the low boiling components, constituting the bulk of the sample, elute. It is possible to achieve a much better separation and by 1968 over 180 components had been separated and identified [1]. However, this analysis employed subambient oven conditions and an analysis time of over 2 h. Complete resolution of such a complex mixture is not possible and so analysts have turned to capillary MDC.

However, there are problems in translating a technique designed on packed columns onto capillary columns. The first problem is that of dead space. Because the flow rate of the carrier gas in capillary GC is very low, typically 0.5–4 ml/min, it is important that there are no unswept volumes or dead space in the system. If there is dead space, then samples can diffuse into it and later diffuse back into the gas stream. This will lead to tailing peaks or ghost peaks appearing in the chromatogram.

Dead space can be a substantial problem in MDC due to the switching system involved. There are two ways of switching the column eluent. It can be either by a pneumatically controlled multiport valve or by the use of a flow-switching technique developed by Deans [2]. With the introduction of zero dead volume valves and unions, either system can be used, but the Deans system is to be preferred since it offers less dead volume and reduced exposure to potentially reactive metal surfaces. It is also easier to set up because it does not require a heated valve.

A further problem, common to all MDC systems, is oven stability and repeatability of retention times. Since a portion of the chromatogram must be cut at exactly the same point in every analysis, it is essential that retention times are consistent. Obviously, oven design is very critical in MDC, particularly with capillary systems and the PU4900 Gas Chromatograph from Phillips employs a revolutionary oven design.

The PU4900 has a 'Low Thermal Lever' (LTL) effect and this is due to careful design of the oven such that the platinum resistance thermometer is situated very close to the column. Consequently, the temperature that is being measured is the actual temperature of the column environment, not the temperature of a part of the oven which is removed from the column. This allows the system to more precisely and more reproducibly follow the temperature program. It also means that if the temperature of the laboratory fluctuates, as it must in a 24 h period, the PRT measures the actual effect of this on the column and can compensate more accurately to maintain the oven set temperature. This effect is easily understood from the graphical representation in figure 3. Figure 4 shows how this has been achieved by the use of the sliding head. This sliding head contains all the detectors, injectors and column couplings ensuring 270° access to the columns.

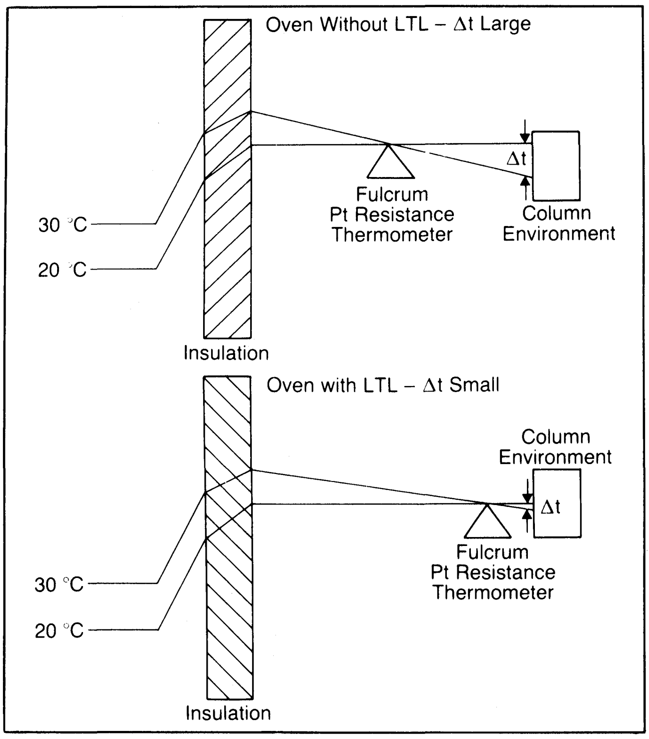

*Figure 3. Low Thermal Lever effect.*

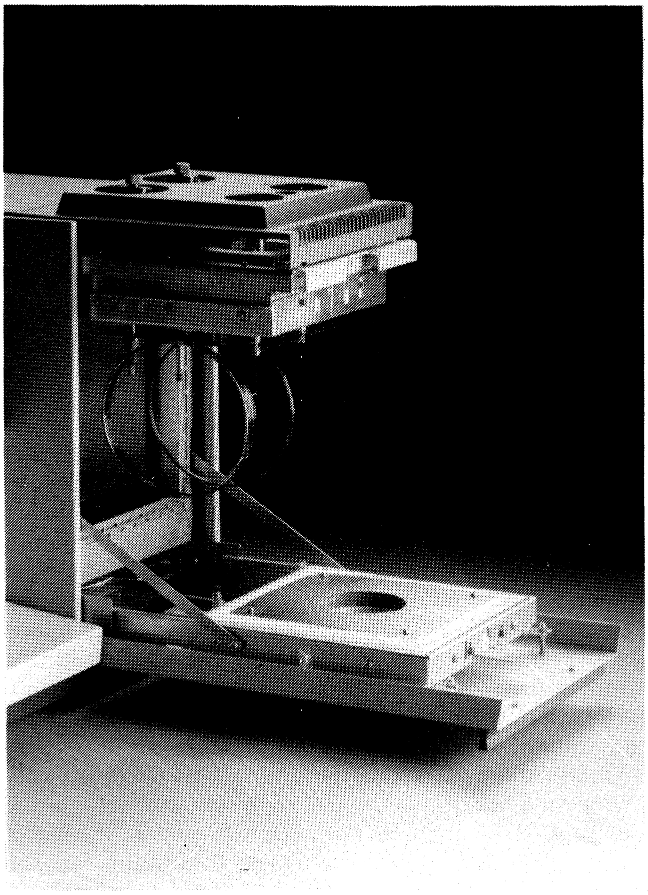

*Figure 4. The PU4900's sliding head.*

However, there is one big disadvantage which until recently has been a major stumbling block in MDC. In conventional oven designs, both of the columns are contained within the same column oven and are subject to the same temperature program. This has been a big limitation and has restricted the potential of capillary MDC systems. However, the PU4900 has got around this problem in a novel and successful way.

In the main instrument the chromatography package is located on the right-hand side of the instrument while the satellite is a 'mirror image' chromatograph and the two ovens can be placed side by side. The ovens can then be linked together in the injection ports, by a temperature controlled interface oven (see figure 5). It is essential that this interface is independently temperature controlled to prevent condensation of sample within the interface.

Although the double oven instrument is not a new idea, what is new is that both ovens are completely separate and that there is no possibility of heat leaking from the hot oven into the cold one via a dividing wall. Both ovens can be temperature programmed independently of each other.

There are many applications of MDC, particularly in the chemical industry, and recently a lot of work has been put into the analysis of oxygenated additives in petrol. These additives are primarily lower alcohols.

For many years, tetraalkyl lead components have been added to petrol to act as 'anti-knocking' agents to prevent pre-ignition. Unfortunately, lead compounds are then vented in the atmosphere where they pose a health problem. There is also a strong environmental, as well as safety, lobby against these lead compounds and the petrol industry has turned to the lower alcohols to act as substitutes. Unfortunately, these alcohols elute from a GC column very quickly and are consequently masked by co-eluting peaks. The problem then is how to identify and quantify the alcohols.

A single capillary column is capable of some resolution, but the analysis is time-consuming and inaccurate. MDC using capillary columns is the only practical means of achieving the desired results.

The system used for the analysis is shown in figure 6 and is a heart cut system. The means of achieving flow switching is based on a Deans system using Valco zero dead volume fittings. The mechanism of operation can be explained with reference to figure 6. The system is set up as follows:

(1) SV and $TV_2$ are closed. $TV_1$ is opened and $PR_1$ adjusted to deliver a suitable flow of carrier gas through $C_1$ and $C_2$ to the detector, D. Once the system has stabilized, the 'mid point' pressure is noted on PG2.

(2) $TV_2$ is opened and $PR_2$ adjusted to give a pressure reading slightly higher than the mid point pressure on PG2 in (1).

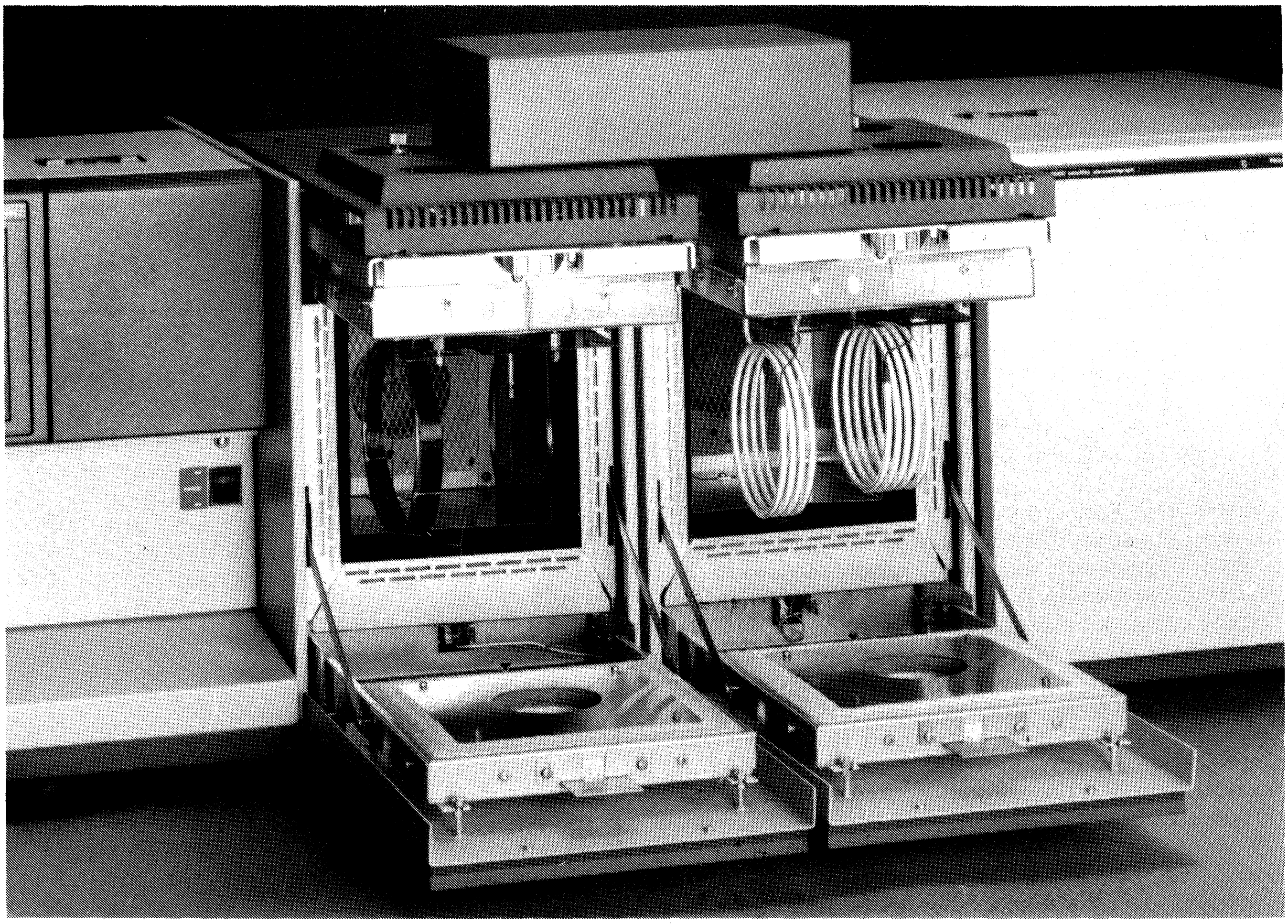

*Figure 5. Oven linking in the PU4900 using a temperature controlled interface oven.*

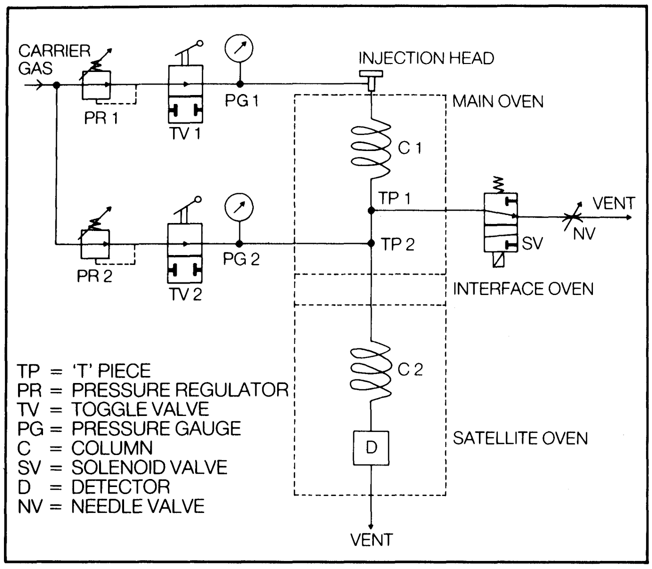

*Figure 6. The heart cut system.*

Care must be taken that the pressure delivered by $PR_2$ is not so great that the pressure differential across the first column, C1, is so low that the carrier gas flow rate in C1 is slow enough for diffusion of the solute peaks to limit the initial separation.

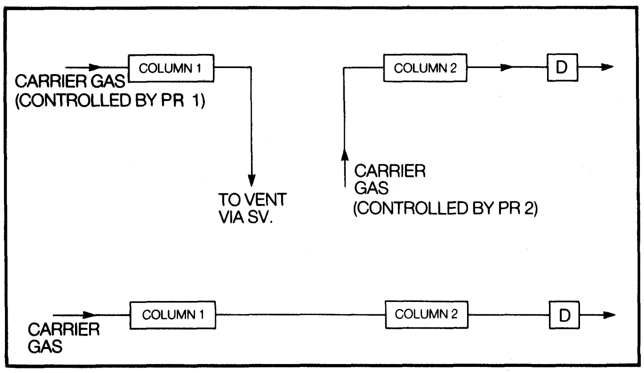

*Figure 7. System operation.*

The system is now ready for operation:

(*a*) When SV is opened the direction of flow is as shown in figure 7. This corresponds to POSITION 1 in figure 1.

(*b*) When SV is closed, the direction of flow is as shown in figure 7 which corresponds to POSITION 2.

All of the Valco zero dead volume fittings are contained in the left-hand oven, i.e. the main instrument oven and by careful design of the path through the interface oven, it is possible to pass a quartz capillary column through the oven without any additional couplings. In order to minimize dead volume and hence peak broadening, it is essential to reduce the number of unions used in the system.

Identification of components is normally by retention time and this is determined by two factors. One is the accuracy and repeatability of the oven controller and the other is the time at which chromatography starts. Oven repeatability has already been discussed in this article; chromatography normally starts at the point of injection. However, in MDC, chromatography on the second column does not start at the same time for all components in the 'heart' and this is the purpose of the cold trap (figure 6).

When the initial injection of sample onto the first column is made, liquid $CO_2$ is squirted into the cold trap in the satellite oven. The purpose of the cold trap is to provide a freezing region so that as material enters the second column, it condenses, freezes and ideally remains in the cold trap until the $CO_2$ is turned off. So, although the 'cutting' time may take several minutes, all of the components in the heart are concentrated in a small region at the front of the column. At the end of the cutting period, the $CO_2$ is turned off and the temperature program started. All material therefore starts chromatography at the same point and the same time, which is equivalent to a syringe injection. Retention times are therefore accurate.

Ideally, all components should be fully trapped in the cold trap and for this to be so, they need to be frozen solid. If they are liquid, they will eventually break through the trap and give diffuse peaks. The cold trap used in this work was supplied by Quadrex and placing a thermocouple on it, a temperature of −60 °C was measured but this is above the true temperature at the centre of the trap by probably 20–30 °C. This is therefore sufficient to trap most compounds, but, if necessary, there are many designs of cold trap using liquid nitrogen at −196 °C.

What then is the sequence of operation? By reference to figure 6:

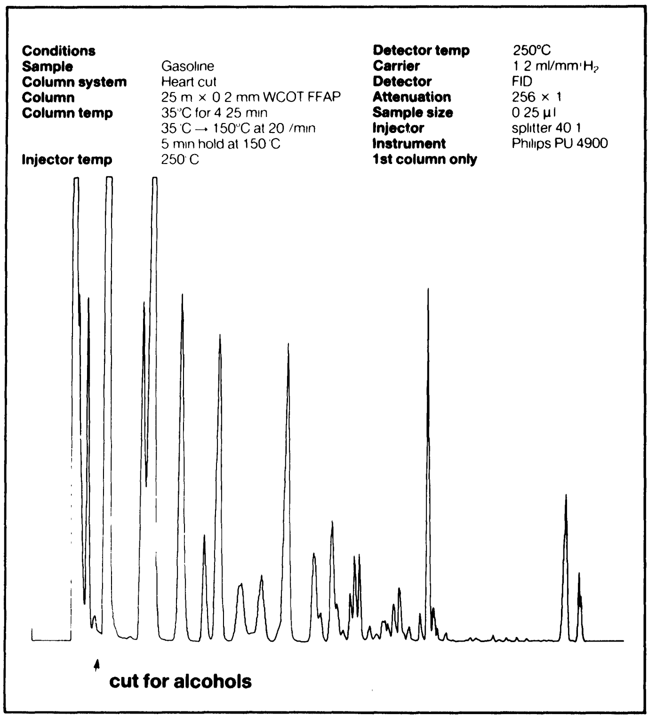

*Figure 8. Petrol on a polar FFAP column—the alcohol region is marked.*

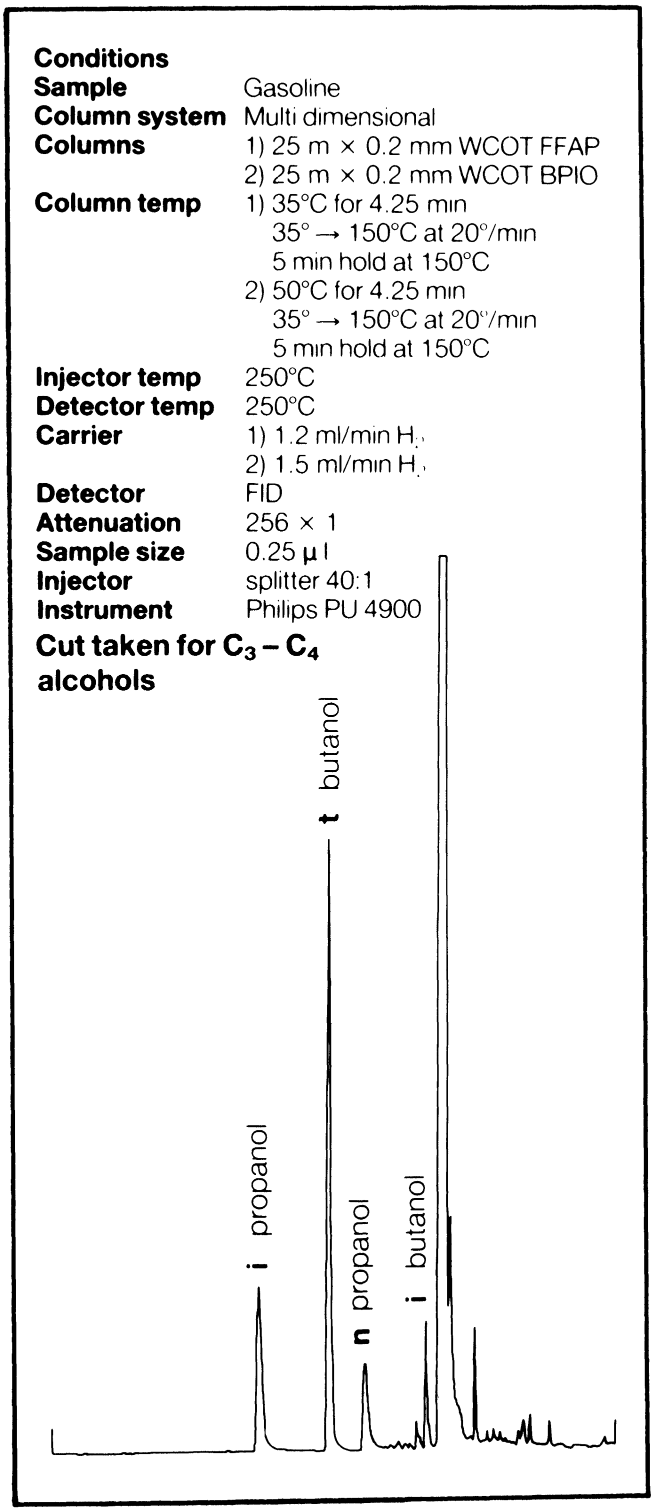

*Figure 9. Petrol sample after cutting.*

(i) A sample is injected with SV open and in this mode, all material eluting from column A is vented to waste. The $CO_2$ is also switched on, in order to drop the temperature of the cold trap.

(ii) After a few minutes, SV is closed and column B is brought in line. Material flows directly from column A to B, through the interface oven and into the satellite oven where it freezes in the cold trap.

(iii) SV is then opened and so column A is open to vent. The oven can be rapidly temperature programmed to remove the higher boiling fraction of the petrol. Meanwhile, in the satellite oven the $CO_2$ has been turned off and an analytical temperature program started. The trapped material from the 'heart cut' begins chromatography and is eventually detected at the FID.

A capillary chromatogram of petrol has already been shown in figure 2 and figure 8 shows the same sample on a polar FFAP column with the alcohol region marked. After cutting, the alcohols are easily identified from figure 9. The analysis time is approximately 10 min.

The reason for using a polar column like FFAP as the initial column is to bunch the alcohols together and separate them from hydrocarbons of similar boiling-point. Consequently, on the medium polarity second column, BP10, where boiling-point is the main separation mechanism, it is easy to separate the alcohols from each other and the few hydrocarbons in the 'heart'.

### Conclusion

An MDC system such as heart cut is a very powerful tool for solving complex analytical problems. The system illustrated here has been used to analyse only a small portion of the total chromatogram in a short time. Using a single column would take about 1 h, so MDC can result in a decrease in analysis time and hence an increase in sample throughput.

Alternatively, a heart cut system which can analyse only a small part of the total chromatogram at a time, but with complete resolution, can be used, by repeated injections, to build up a complete picture of the sample. Such an analysis would obviously be tedious and time-consuming and it is in this field that the true integrated power of the PU4900 can be demonstrated.

The PU4900's sequence table controlling the PU4700 autojector can be set such that each sample can have a different analytical method. Each method is a complete method and contains details of temperature programs, timed events for switching the Deans system, calculation, peak tables etc. It is therefore possible to envisage a completely automatic system which on the first injection cuts the first 30s of chromatogram onto the second column for resolving. In the second injection, the cut period is now 30s to 60s; the third injection cuts from 60s to 90s. As the fractions cut onto the second column, increase in boiling-point, the temperature program advances in order to achieve resolution.

The data resulting from these multiple analyses can be quantified after identification using the PU4900 software or if the identity is unknown then the results table can be output via the RS232C interface to an external computerized retention index library. In this way full identification and quantitation of a complex sample is possible.

### References

1. *Chromatographia*, **1** (1968), 18.
2. *Journal of Chromatography*, **18** (1965), 477.

**FLOW ANALYSIS III**

The Third International Conference on Flow Analysis will be held in Birmingham, UK from 5 to 8 September 1985, organized by the Midlands Region of the Analytical Division of the Royal Society of Chemistry. The scope of the conference will be similar to the previous Flow Analysis Meetings (in Amsterdam in 1979 and in Lund in 1982) and current research on all aspects of continuous flow analysis will be covered, including:

Instrumentation for flow injection analysis and for continuous segmented and unsegmented flow analysis, and approaches to total automation; New detector systems and hybrid systems; Theory of flow analysis; Applications in industrial, environmental and clinical analysis.

Invited lecturers include Dr Stewart, Professors Riley and Ruzička. The scientific programme is being arranged by Dr A. M. G. Macdonald, Department of Chemistry, The University, PO Box 363, Birmingham B15 2TT, UK.

*Registration forms are available now from The Secretary, Analytical Division, Royal Society of Chemistry, Burlington House, London W1V 0BN.*

